# Genome-Wide Characterization of the *MDS* Gene Family in *Gossypium* Reveals *GhMDS11* as a Key Mediator of Cold Stress Response

**DOI:** 10.3390/ijms262010144

**Published:** 2025-10-18

**Authors:** Xuehan Zhu, Ahmad Haris Khan, Yihao Liu, Allah Madad, Faren Zhu, Junwei Wang, Ganggang Zhang, Fei Wang, Zihan Li, Shandang Shi, Hongbin Li

**Affiliations:** 1 Key Laboratory of Oasis Town and Mountain-Basin System Ecology of Xinjiang Production and Construction Corps, Key Laboratory of Xinjiang Phytomedicine Resource and Utilization of Ministry of Education, College of Life Sciences, Shihezi University, Shihezi 832003, China; xuehan_zhu@foxmail.com (X.Z.); hariskhan@stu.shzu.edu.cn (A.H.K.); yihao0606l@163.com (Y.L.); madadnewchina@stu.shzu.edu.cn (A.M.); zhufaren163@163.com (F.Z.); 18287018531@163.com (J.W.); 19915233386@163.com (G.Z.); feiw@shzu.edu.cn (F.W.); 2 Department of Civil, Environmental and Construction Engineering, College of Engineering and Computer Science, University of Central Florida, Orlando, FL 32816, USA; zihl2721@gmail.com

**Keywords:** cotton, *GhMDS* gene family, cold tolerance, virus-induced gene silencing, transcriptome

## Abstract

Cotton’s susceptibility to low temperatures makes it a crucial raw resource for the world’s textile industry, yet its cultivation in temperate regions is severely limited. Although plant growth and stress responses depend on receptor-like kinases (RLKs), the functions of the *MEDOS* (*MDS*) gene family, which includes genes that encode RLK, are still poorly understood in cotton. In this study, we conducted a genome-wide analysis to systematically investigate the distribution of *MDS* gene family members in four cotton species. Phylogenetic analysis identified five evolutionary clades of the *MDS* gene family in cotton. The role of promoter *cis*-acting elements in hormone signaling and abiotic stress responses was suggested by analysis. Collinearity analysis demonstrated that segmental duplication was the primary driver of family expansion. Gene expression profiling showed that *GhMDS11* was significantly upregulated under cold stress. Functional validation through silencing *GhMDS11* compromised cold tolerance, confirming its role in stress adaptation. Comparative transcriptome study of silenced plants demonstrated substantial enrichment in pathways associated with hormone signal transduction and fatty acid breakdown. It is speculated that the chain of “hormone synthesis → signal transduction → secondary metabolism” completely presents the transcriptional regulation network and functional response of plants after receptor kinase VIGS. Silencing the *GhMDS11* gene in cotton initiates regulatory effects through hormone synthesis, which is amplified via a signal transduction cascade, ultimately affecting secondary metabolism. This comprehensive pathway clearly demonstrates the downstream transcriptional reprogramming and functional changes. This work thoroughly examined the evolutionary traits of the *MDS* family across four cotton species and clarified the functional and molecular processes of *GhMDS11* in improving low-temperature tolerance, laying a solid foundation for further clarifying multidimensional regulatory networks and breeding cold-resistant cotton materials. Simultaneously, our findings pave the way for future research to develop molecular markers, which could potentially shorten the breeding cycle and facilitate the targeted enhancement of cold tolerance in cotton.

## 1. Introduction

Cotton is a warm season crop native to tropical and subtropical regions [1,2]. Despite extensive domestication and selective breeding, cotton remains highly sensitive to low-temperature stress, a vulnerability deeply rooted in its evolutionary background [3,4,5]. In China’s cotton-producing regions, persistently low and unstable spring temperatures subject cotton seedlings to unfavorable growing conditions [6,7]. This not only leads to stunted growth and seedling mortality but ultimately reduces cotton yield [8,9]. With increasing climatic variability and the expansion of cotton cultivation into temperate zones, enhancing cold resistance has become a priority in breeding programs [9,10,11,12]. Current research endeavors concentrate on amalgamating cold endurance with elevated yield and exceptional fiber quality to tackle the problems presented by more erratic environmental circumstances [13,14,15]. This emphasis on cold resistance not only safeguards cotton productivity but also ensures a stable supply for the global textile industry, highlighting its dual significance in both agriculture and economic sustainability [16,17]. Therefore, a deeper understanding of the molecular mechanisms underlying cotton’s cold tolerance holds substantial importance for both biological research and agricultural production [18].

The *MDS* gene family, an important group of genes encoding RLKs in plants, has become a focal area of functional research due to its critical role in regulating immune responses and stress tolerance. First identified in *Arabidopsis thaliana*, the *MDS1*, *MDS2*, *MDS3*, and *MDS4* genes belong to the Malectin_like superfamily. In *A. thaliana*, *MDS1* is implicated in primary root development, while *AtMDS* is engaged in ABA-regulated stomatal migration. *AtMDS1* and *AtMDS2* serve as core components of the SUMM2-mediated immune system [19]. Specifically, AtMDS1 interacts with AtCRCK3 in plants, leading to its phosphorylation, which stabilizes SUMM2 activation and amplifies immune responses, thereby positively regulating SUMM2-mediated pathogen defense mechanisms [19]. The WRKY33 transcription factor interacts directly with the promoter regions of *MDS1* and *MDS2*, upregulating *MDS* expression in association with a comprehensive defense network. It also alters the interaction between *Pseudomonas syringae* and necrotic fungal pathogens [20,21]. Additionally, *MDS* genes participate in a complex network involving CrRLK1L, where they can either positively or negatively regulate plant growth. These findings underscore how *MDS* genes are integrated into intricate signaling pathways to enhance plant stress resistance. Significant progress has been made in elucidating plant response mechanisms to cold stress, including cold acclimation processes and the roles of various gene families [22,23]. However, the specific functions of *MDS* genes from the RLK family in cotton’s cold tolerance remain poorly understood.

This work offers a comprehensive examination of the *MDS* gene family’s genomic traits and functional roles in cotton, emphasizing its crucial role in cold adaptation. By conducting a comprehensive genome-wide investigation, we discovered many *MDS* homologous genes in cotton and clarified their evolutionary links, structural diversity, and regulatory factors. Transcriptome study indicated that some *MDS* genes in cotton leaves exhibited considerable upregulation during cold stress, suggesting these genes are crucial regulatory elements in temperature response networks. To elucidate the functional importance of these genes, we amalgamated single-gene association research data from *Gossypium hirsutum*, revealing that certain *MDS* members have a strong link with improved cold resistance in cotton. Functional validation by virus-induced gene silencing (VIGS) technology showed that the targeted suppression of critical candidate *MDS* genes significantly diminished cold tolerance in cotton plants. In the future, we will study the complete pathway of *MDS* gene involved in cotton cold stress according to the content of this study, which not only provides the molecular basis for cotton cold stress signal pathway, but also provides a theoretical basis for practical application.

## 2. Results

### 2.1. Identification, Chromosomal Localization, and Physicochemical Property Analysis of the MDS Gene Family

**Figure 1:**
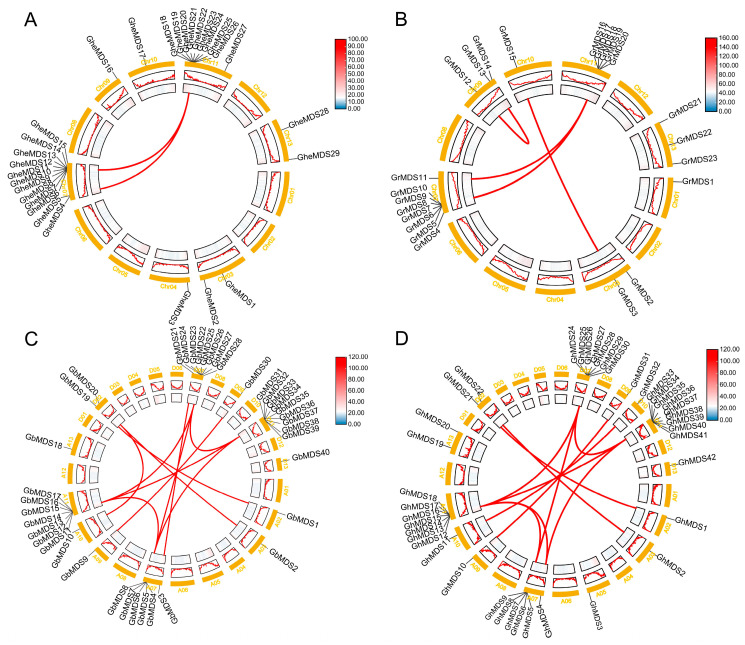
Chromosomal location and collinearity of the *MDS* gene family in four *Gossypium* species. (**A**–**D**) The chromosomes of *G. herbaceum*, *G. raimondii*, *G. barbadense*, and *G. hirsutum*.

We discovered 42 non-redundant MDS protein genes in *G. hirsutum*, 29 MDS protein genes in *Gossypium herbaceum*, 23 in *Gossypium raimondii*, and 40 in *Gossypium barbadense* using genome-wide analysis using HMMER software (v3.4), which was verified by Pfam and InterProScan. According to their chromosomal locations, the genes were systematically designated as *GheMDS1–GheMDS29*, *GrMDS1–GrMDS23*, *GhMDS1–GhMDS42*, and *GbMDS1–GbMDS40*, respectively (Figure 1 and Table S2). In *G. hirsutum*, these genes were distributed across all 15 chromosomes: one member each was located on chromosomes A02, A03, A05, A09, A10, D04, D09, D10, and D13, while the remaining chromosomes contained multiple members. The distribution pattern of the *MDS* gene family in *G. barbadense* was similar to that in *G. hirsutum*. In *G. herbaceum*, the *MDS* gene family was primarily distributed on chromosomes 7 and 11, while in *G.raimondii*, it was mainly located on chromosome 11.

Analysis of the physicochemical properties of GhMDS genes revealed significant diversity among these proteins (Table S1): The quantity of amino acid residues among family members varies significantly, with molecular weight trends consistent with amino acid length, indicating substantial variation in the primary structure (amino acid composition and quantity) of this protein family, which may be related to functional differentiation or differences in subcellular localization. The isoelectric point ranges from 4.89 to 7.19, showing an overall acidic or neutral tendency. Typically, proteins with instability indices > 40 are more unstable in vitro and prone to degradation, while those <40 are relatively stable. Within this family, some members exhibit higher stability and may be suitable for long-term structural or catalytic functions; others show lower stability and may function as short-term regulatory proteins (such as temporary signaling molecules) that require rapid synthesis and degradation to respond to environmental changes. The percentage of aliphatic amino acids (such alanine, valine, and leucine) in the protein is indicated by the aliphatic index, which varies from 77.65 to 93.48. Higher values indicate a greater proportion of hydrophobic amino acids, suggesting that the protein may more readily form hydrophobic cores or bind to membrane structures. The GRAVY score ranges from −0.292 to 0.042, and virtually all MDS proteins are scored negatively, so most MDS proteins are hydrophilic (positive GRAVY values denote hydrophobicity, whereas negative values denote hydrophilicity). This suggests that the proteins in this family are generally hydrophilic or moderately hydrophobic, which may be related to hydrophobic domains participating in protein–protein interactions. The physicochemical properties of this protein family (length, molecular weight, charge, stability, hydrophobicity) exhibit significant diversity, reflecting variations in primary structure and surface characteristics. This variation may stem from the prolonged evolutionary adaptation of family members to varying functional demands. Subcellular localisation predictions indicate that GhMDS proteins are primarily located in the plasma membrane.

### 2.2. Analysis of Gene Duplication and Evolutionary Selection Patterns in the MDS Gene Family

**Figure 2:**
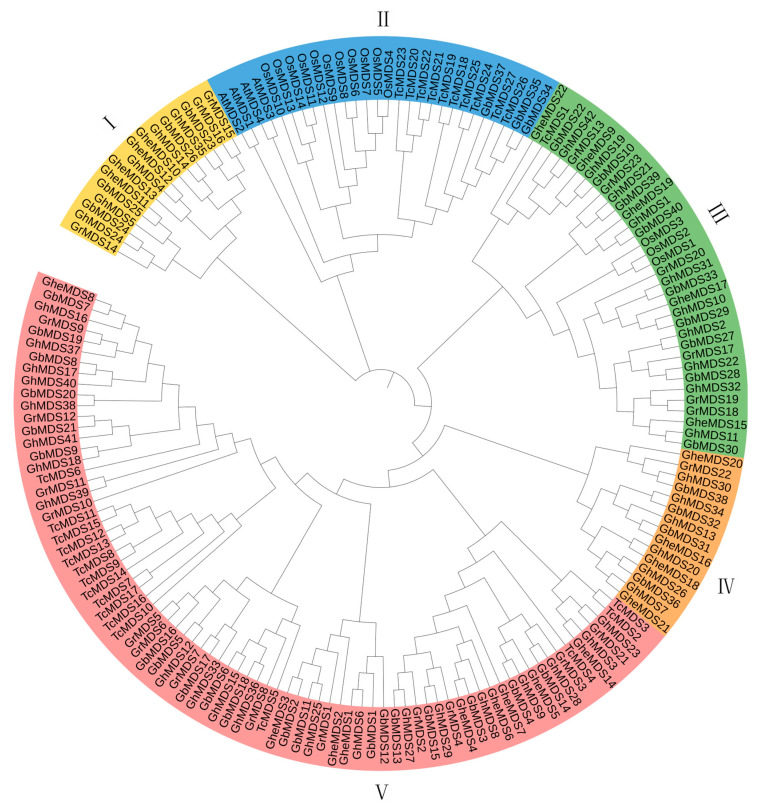
MDS protein in *A. thaliana*, four *Gossypium* species, *O. sativa*, and *T. cacao*. Maximum Likelihood was used to create the phylogenetic tree (ML). The ML tree’s branches are color-coded based on their membership in sub-families, with each color represented by a Roman numeral indicating its corresponding group.

We investigated the evolutionary pathways of the *MDS* gene family by reconstructing a phylogeny with protein sequences from seven species (Figure 2). The species analyzed were: *A. thaliana* (At, 4), *G. barbadense* (Gb, 40), *G. hirsutum* (Gh, 42), *Theobroma cacao* (Tc, 27), *Oryza sativa* (Os, 14), *G. raimondii* (Gr, 23), and *G. herbaceum* (Ghe, 29). The research demonstrated that these *MDS* genes grouped into five separate branches. Some *MDS* genes of *O. sativa*, all *MDS* genes of *A. thaliana*, and some *MDS* genes of *T. cacao*, along with three *MDS* genes of *G. barbadense*, were positioned in the II evolutionary branch. This suggests that the rapid expansion of *MDS* genes may have occurred after the divergence of monocotyledons and dicotyledons. The MDS proteins of *O. sativa* were primarily clustered in the II branch, with a minority (3) distributed in the III branch. *TcMDS* genes were mainly found in the II branch (10) and V branch (16). The V branch exclusively contained cotton *MDS* genes apart from those of *T. cacao*. The IV and I evolutionary branches consisted entirely of cotton *MDS* genes, while the III branch, besides cotton *MDS* genes, included only one *TcMDS* gene and three *OsMDS* genes. This distribution pattern indicates that *A. thaliana* and *O. sativa* exhibit unique evolutionary conservation trajectories compared to cotton *MDS* homologous genes. Overall, these results reveal divergent evolutionary patterns and varying degrees of sequence conservation among *MDS* genes in different plant species.

To elucidate the driving mechanisms of *MDS* gene families, researchers analyzed *MDS* gene duplication events in four cotton varieties. Gene pairs identified through co-linear analysis were visualized using circular diagrams (Figure 1). Comparative analysis of co-linear results between tetraploid and diploid cotton varieties revealed that these cotton varieties collectively generated 29 gene pairs through *MDS*. This suggests that the evolution and expansion of *MDS* gene families in cotton are primarily driven by fragment duplication events. The findings indicate that fragment duplication may serve as the primary driving force behind *MDS* gene amplification in cotton.

### 2.3. Gene Structure Analysis of the MDS Gene Family

**Figure 3:**
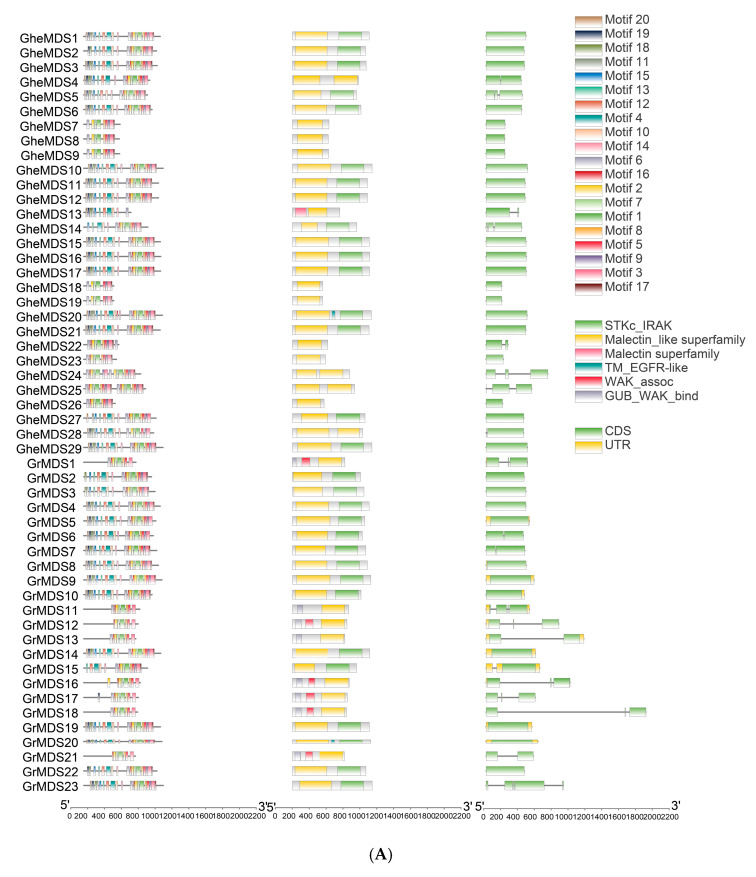

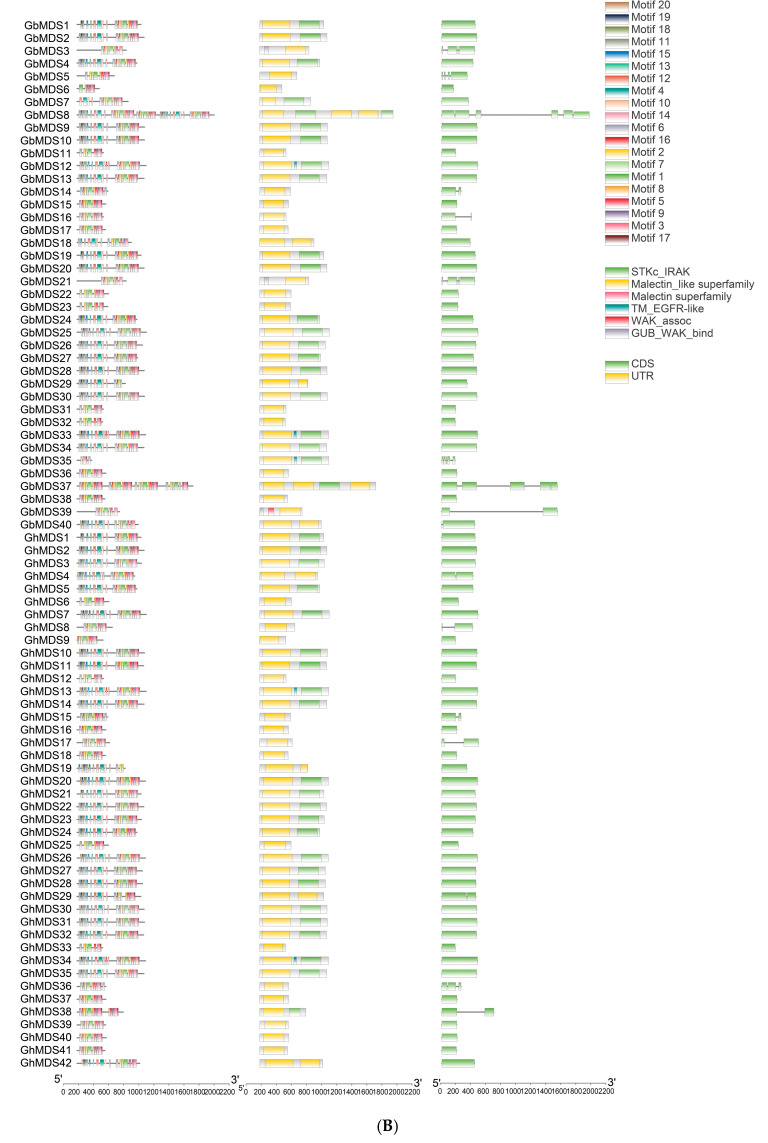
(**A**). Sequence attributes of *MDS* genes among *G. herbaceum* and *G. raimondii*. Ghe is *G. herbaceum.* Gr is *G. raimondii.* (**B**). Sequence attributes of *MDS* genes among *G. barbadense* and *G. hirsutum.* Gb is *G. barbadense*. Gh is *G. hirsutum*.

Gene structure analysis further confirmed these evolutionary relationships, with conserved exon-intron patterns being observed within each clade. Structural analysis of *MDS* genes in four *Gossypium* species was performed using TBtools (v2.210) (Figure 3), and the results demonstrated relatively conserved gene structures among *MDS* family members. In *G. hirsutum*, *MDS8*, *MDS15*, *MDS4*, *MDS17*, *MDS29*, *MDS36*, and *MDS38* contained intron structures. In *G. barbadense*, *GbMDS3*, *GbMDS5*, *GbMDS14*, *GbMDS16*, *GbMDS21*, *GbMDS35*, *GbMDS37*, and *GbMDS39* possessed intron structures. In *G. herbaceum*, *GheMDS4*, *GheMDS13*, *GheMDS22*, *GheMDS24*, *GheMDS25*, and *GheMDS28* contained introns, while in *G. raimondii*, half of the members had intron structures. The gene structures were relatively simple.

UsingMEME Suite online platform, conserved motifs of *MDS* family members were predicted, resulting in the identification of 20 motifs designated as Motif 1 to Motif 20. Among these, every gene contained Motif 2, and 41 family members (except *MDS9*) contained Motif 6 and Motif 12. At least eight conserved motifs were present in each member of the family: Motif 1–4, Motif 7, Motif 9, Motif 17, and Motif 18. Some family members contained 9 to 20 motifs, indicating that the functions of the 42 *GhMDS* family members were relatively conserved. The conservation of these structural features across different *Gossypium* species suggests evolutionary maintenance of critical functional domains, while variations in motif composition may reflect functional diversification among specific gene family members. The presence of consistent core motifs alongside variable accessory motifs provides insights into both conserved molecular functions and potential specialized roles developed during cotton evolution.

### 2.4. Prediction and Analysis of Promoter Function in the MDS Gene Family

**Figure 4:**
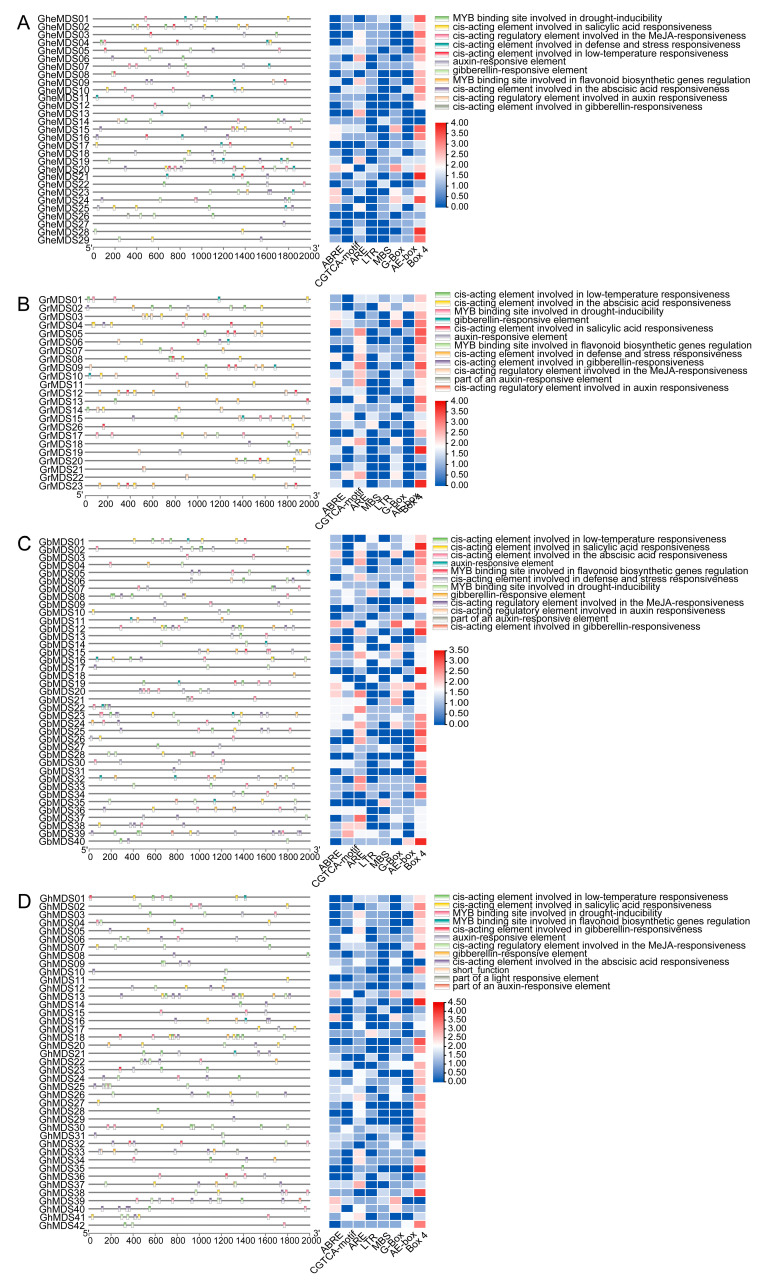
Examination of the kinds and concentrations of *cis*-acting elements found inside the *MDS* gene family’s promoters. Following Log2 transformation, the number of *cis*-acting elements was shown. (**A**–**D**) Analysis of the kinds and concentrations of *cis*-acting elements in *G. herbaceum*, *G. raimondii*, *G. barbadense*, and *G. hirsutum*’s *MDS* gene family promoters. Both the CGTCA motif and the ABRE motif respond to hormones. A stress response factor is what ARE does. Low-temperature-responsive *cis*-acting elements, LTR and MBS, function as MYB binding sites linked to drought induction, respectively. Light-responsive elements are G-box, AE-box, and Box4.

Examination of the *MDS* gene promoters in four *Gossypium* species showed that their *cis*-acting elements are largely responsible for four functions: responding to hormones, responding to stress, regulating by light, and governing growth and development (Figure 4). The ubiquitous presence of light response elements in all promoters shows their conserved function in light regulation. Hormone and abiotic stress-related *cis*-elements, including those responsive to auxin, were abundantly distributed in the promoters of *GhMDS* genes (*GhMDS2*, *GhMDS3*, *GhMDS6*, *GhMDS12*, *GhMDS15*, *GhMDS17*, *GhMDS21*, *GhMDS23*, *GhMDS25*, *GhMDS33*, *GhMDS36*, *GhMDS39*), gibberellin response elements (*GhMDS1*, *GhMDS18*, *GhMDS23*, *GhMDS32*, *GhMDS36*, *GhMDS5*, *GhMDS12*, *GhMDS13*, *GhMDS16*, *GhMDS20*, *GhMDS22*, *GhMDS33*, *GhMDS34*, *GhMDS37*). Among the *GhMDS* genes, 28 possessed ABA response elements in their promoter regions, with methyl jasmonate response elements detected in 23. Promoters from 23 *GhMDS* genes contained *cis*-elements for both drought and low-temperature response, pointing to their potential in mediating stress adaptation. The comprehensive distribution of these regulatory elements across different *GhMDS* promoters indicates complex transcriptional regulation mechanisms that may coordinate hormonal signaling and stress responses in cotton, with particular enrichment of abiotic stress-related elements suggesting specialized adaptation to environmental challenges. The presence of multiple hormone response elements within distinct promoters indicates potential interactions among different phytohormone signaling pathways in the control of *MDS* gene expression.

### 2.5. Expression Profiles of GhMDS Genes in Different Tissues and Under Various Stress Conditions

**Figure 5:**
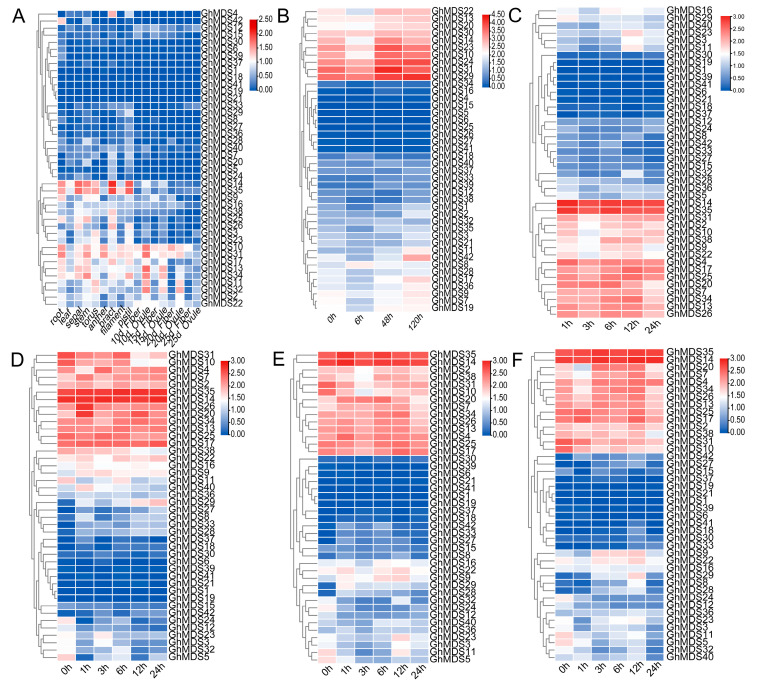
Schematic representation of the expression patterns of the G. hirsutum *MDS* gene across several tissues and in response to stress treatments. (**A**) Analysis of *GhMDS* expression profiles across several tissues. (**B**–**F**) Analysis of *GhMDS* expression patterns following *V. dahliae* infection. *GhMDS* gene family expression profiles at 0 h, 1 h, 3 h, 6 h, 12 h, and 24 h under drought stress (PEG treatment), salinity stress (NaCl treatment), cold stress (4 °C treatment), and heat stress (37 °C treatment).

This study investigated the spatiotemporal expression patterns of 42 *GhMDS* genes across diverse organs and under varied stress circumstances to clarify their biological activities. Analysis of the expression profiles of *GhMDS* family members in different tissues (Figure 5A) showed that some *GhMDS* genes were usually expressed at higher levels in vegetative and reproductive tissues. Especially, *GhMDS10* displayed exceptionally strong expression characteristics in roots, while *GhMDS14* showed predominant expression in stems and anthers, suggesting these genes might be involved in lignin biosynthesis or nutrient transport processes. The overall suppression in reproductive tissues reflects the preferential allocation of metabolic resources toward vegetative growth during cotton development. The *GhMDS* gene family demonstrated distinct temporal expression patterns in response to diverse stress conditions. Under drought stress, the expression patterns of *GhMDS31*, *GhMDS2*, *GhMDS10*, and *GhMDS38* showed consistent changes: their expression levels decreased at 3 h but increased during other periods. In contrast, *GhMDS11* exhibited distinct dynamics: its expression rose between 1 h and 12 h under drought stress, followed by a decline between 3–6 h and 24 h (Figure 5C). *GhMDS35* exhibited prompt early induction under osmotic stress. In response to salt stress (Figure 5D), the *GhMDS35* gene is immediately activated and maintains continuous expression for 1–24 h. Meanwhile, *GhMDS7* and *GhMDS20* show increased expression at 1 h followed by subsequent decrease. Regarding temperature stress responses, *GhMD11* exhibits similar patterns of expression in both cold and heat stress conditions. During cold stress (Figure 5E), *GhMDS11* demonstrates a sustained decline in expression from 1 to 6 h, followed by an increase at 12 h and a decrease at 24 h. After heat treatment (Figure 5F), *GhMDS11* shows continuous reduction in expression from 3 h onward, with a rebound between 6 and 12 h and a final decrease at 24 h. Transcriptome analysis of cotton plants infected with *Verticillium dahliae* demonstrated that *GhMDS22*, *GhMDS13*, *GhMDS30* and *GhMDS31* exhibited strong upregulation during late infection stages (Figure 5B). Cross-analysis of phylogenetic and expression data revealed that genes within the same clade often exhibited similar expression patterns. Specifically, the III clade containing *GhMDS11* (Figure 2) included several members (e.g., *GhMDS22*, *GhMDS31*) that were highly change under both cold stress and *V. dahliae* infection (Figure 5). Promoter analysis further indicated that these co-expressed genes commonly possessed stress-related cis-elements, such as the low-temperature-responsive LTR (Figure 4). These observations suggest that evolutionary relatedness within this clade is associated with a coordinated role in stress response. Regardless of stress type, *GhMDS14* consistently maintained high expression levels. The varied expression patterns indicate functional specialization within the *GhMDS* family, with certain genes responding to multiple stresses and others exhibiting stress-specific regulation, demonstrating their adaptation to diverse environmental challenges during cotton growth and development. The tissue-specific expression profiles suggest potential roles in organ development and physiological processes, with notably robust expression in roots and stems indicating significance in abiotic stress responses and structural development.

### 2.6. Functional Validation Analysis of GhMDS Gene Transcriptomes

**Figure 6:**
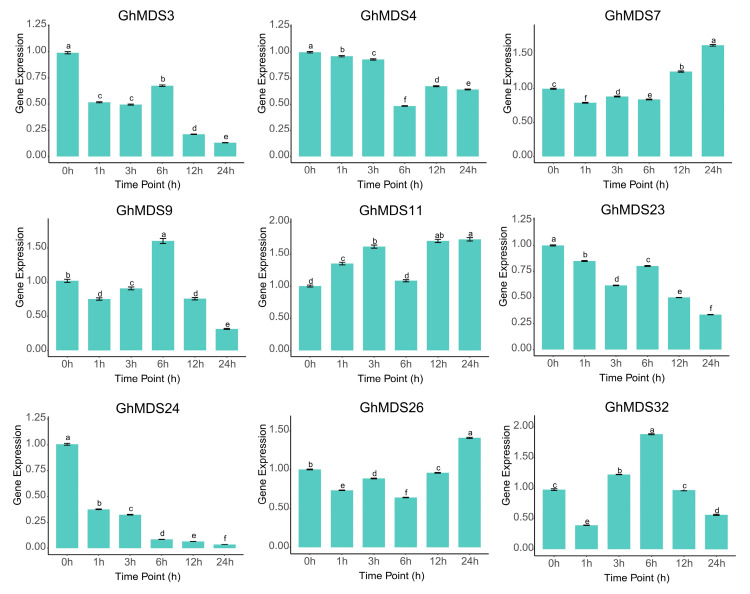
qRT-PCR validation of relative expression of partial *MDS* family genes in *G. hirsutum*. Different letters at various time points indicate statistically significant differences in relative expression levels among groups. Data are presented as mean ± SD (*n* = 3). Different lowercase letters above the bars indicate statistically significant differences based on one-way ANOVA followed by Tukey’s test (*p* < 0.05).

The integration of RNA-seq and RT-qPCR exhibited complementarity: RT-qPCR offered accurate quantification for low-abundance transcripts, whereas RNA-seq encompassed genome-wide expression dynamics. The nuanced discrepancies in absolute expression levels between the two approaches may stem from technical disparities in normalization procedures and sensitivity. Selected *GhMDS* genes under cold stress (*GhMDS32*, *GhMDS26*, *GhMDS24*, *GhMDS23*, *GhMDS11*, *GhMDS9*, *GhMDS7*, *GhMDS4*, and *GhMDS3*) were validated at the expression level based on transcriptome data of the *GhMDS* gene family under cold stress (Figure 6). The results aligned with the transcriptome findings, validating the dependability of both analytical methods. This dual-method validation strategy not only cross-verified the expression patterns but also highlighted the technical characteristics of each platform, with RT-qPCR’s superior sensitivity for low-expression genes complementing RNA-seq’s comprehensive profiling capability. The agreement between these separate approaches enhances the reliability of the identified cold-responsive expression patterns across *GhMDS* family members, establishing a robust basis for future functional research of these candidate genes in cotton’s cold stress response pathways.

### 2.7. GhMDS11-Silenced Cotton Plants Exhibit Increased Sensitivity to Cold Stress

**Figure 7:**
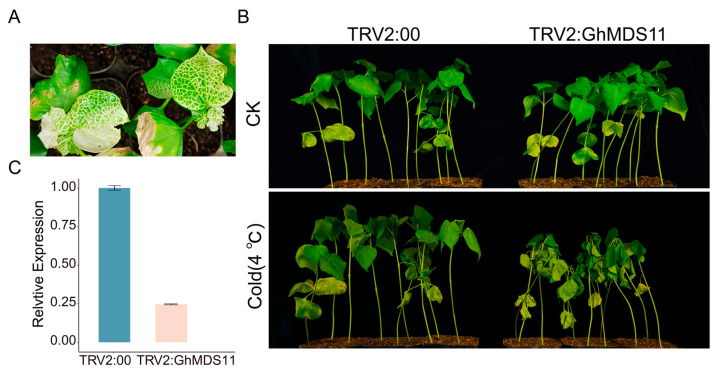
Impact of VIGS-mediated *GhMS11* silencing on cotton’s ability to withstand cold. (**A**) Phenotype of positive control *TRV2:PDS*; (**B**) Phenotype of *GhMDS11*-silenced *G. hirsutum* plants after cold stress treatment; (**C**) RT-qPCR analysis of *GhMDS11* gene expression level changes in cotton leaves.

The heatmap analysis of the *GhMDS* gene family under various biotic and abiotic challenges indicated that *GhMDS11* exhibited the most pronounced variations in expression across diverse stress conditions. This is the rationale for its selection in functional validation for cold stress response. This research utilized VIGS technology to create *GhMDS11*-silenced plants (*TRV2:GhMDS11*), employing empty vector controls (*TRV2:00*) for functional validation. Ten days post-inoculation, the positive control plants (*TRV2:GhPDS*) exhibited distinct leaf bleaching phenotypes, thereby validating the efficacy of the VIGS system. Subsequently, RT-qPCR analysis of *GhMDS11* silencing efficiency revealed significantly reduced relative expression levels of *GhMDS11* in *TRV2:GhMDS11* plants compared to controls, demonstrating successful *GhMDS11* silencing. The silenced plants (*TRV2:GhMDS11*) showed significant wilting, leaf desiccation, and curling signs after being exposed to 4 °C cold stress for 48 h. The control plants (*TRV2:00*) showed less damage (Figure 7). These results demonstrate that silencing *GhMDS11* significantly compromises cotton’s tolerance to low-temperature stress, GhMDS11 may be a candidate gene as a key genetic determinant of cold stress response in upland cotton. The phenotypic severity correlated with molecular silencing efficiency, showing a clear genotype-phenotype relationship in cold stress susceptibility. This functional evidence complements the expression profile data, providing mechanistic insights into *GhMDS11*’s role in cotton’s cold adaptation.

Under 4 °C low-temperature stress (lasting 48 h), *GhMDS11*-silenced plants exhibited significant physiological changes compared to the control group (pTRV empty vector). The silenced plants showed substantial reductions in superoxide dismutase (SOD), catalase (CAT), and peroxidase (POD) activities, indicating impaired reactive oxygen species scavenging capacity and osmotic regulation function. Concurrently, malondialdehyde (MDA) levels were significantly elevated in silenced plants (Figure S1).

### 2.8. Comparative Transcriptome Analysis of GhMDS11-Silenced Cotton Plants Through GO Enrichment Analysis

To investigate the regulatory mechanism of *GhMDS11* in cotton’s cold stress response, researchers conducted RNA-seq analysis on *TRV2:GhMDS11* gene-silenced plants and the control group (*TRV2:00*). Applying a threshold of |log_2_FC| ≥ 1 and FDR < 0.05, we identified 4103 differentially expressed genes (DEGs), comprising 1683 upregulated and 2420 downregulated transcripts (*p* < 0.05). The DEGs were categorized into BP, MF, and CC domains and significantly enriched in 884 GO terms. Within BP, they were predominantly associated with responses to chitin, jasmonic acid, wounding, light stimuli, and drugs, indicating a concerted role in organismal defense pathways.

**Figure 8:**
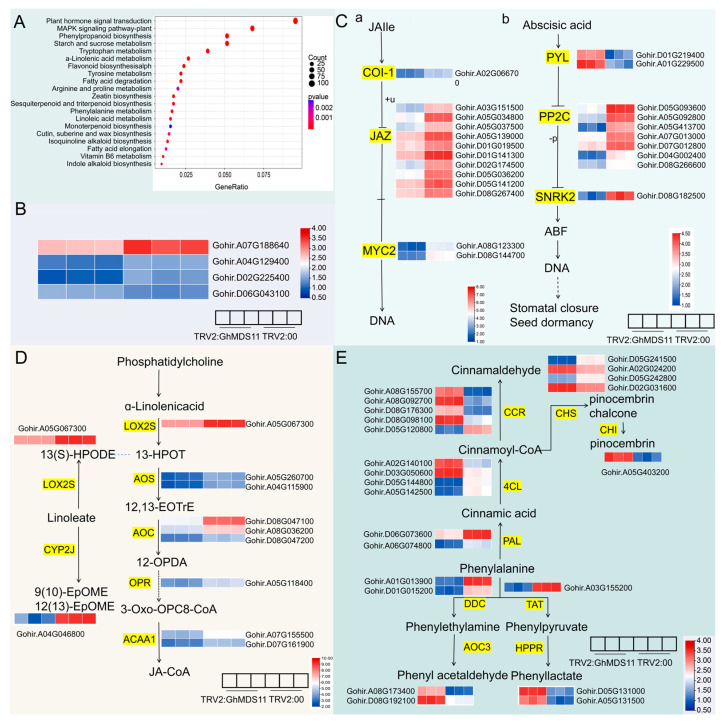
Alterations in Metabolic Transcriptomes upon *GhMDS11* Silencing in Cotton. (**A**) A bubble chart that shows pathways that are significantly more common in the KEGG annotation analysis of genes that are expressed differently. (**B**) Expression of cold stress-related genes in differential genes. (**C**) JA and ABA signal transduction. (**D**) α-linolenic acid metabolic pathway. (**E**) Benzopyrene and flavonoid biosynthesis pathway. The highlighted content refers to key genes or proteins in the pathway.

At the cellular component level, the DEGs were primarily localized in key regions such as the intrinsic components of the plasma membrane, the apoplast, the integral components of the plasma membrane, the chromosomal passenger complex, and the 3-methyl-2-oxobutanoate dehydrogenase (lipoamide) complex. These genes may play critical roles in cell mitosis and the transport of substances within plant cells. To understand the biological mechanisms underlying the cotton differentially expressed genes (DEGs), we performed KEGG annotation analysis. Based on the KEGG annotation, all DEGs were mapped to five primary categories: metabolism, environmental information processing, cellular processes, genetic information processing, and BRITE hierarchies. Among these, metabolism and BRITE hierarchies were the most abundantly annotated categories. The metabolic pathways of primary metabolism included energy metabolism, glycan biosynthesis, lipid metabolism, and nucleotide metabolism. In contrast, the metabolic pathways related to secondary metabolism primarily involved the biosynthesis of other secondary metabolites, as well as xenobiotic biodegradation and metabolism, and the metabolism of terpenoids and polyketides. KEGG enrichment analysis was performed on the DEGs, with a significance threshold set at *p* < 0.05, and the top 20 significantly enriched metabolic pathways were selected to generate a bubble plot for visualization (Figure 8A). By comparing the transcriptome differential genes, a total of 37 metabolic pathways were enriched, with 20 pathways showing significant enrichment. There were several biosynthesis and signaling pathways that the differentially expressed genes were primarily classified under: the plant MAPK signaling pathway, the plant hormone signal transduction pathway, fatty acid degradation, α-linolenic acid metabolism, flavonoid biosynthesis, cutin, suberine, wax biosynthesis, and zeatin biosynthesis. In the JA biosynthesis pathway, the DEGs included genes such as *LOX2S*, *AOS*, *AOC*, *OPR*, *ACAA1*, *TIFY*, the JA receptor Coronatine Insensitive 1 (*COI1*), and *MYC2*. Silencing the *GhMDS11* gene not only affected JA biosynthesis but also disrupted JA signal transduction (Figure 8C-a). *PP2C* is a key player in ABA signal transduction, and silencing the *GhMDS11* gene reduced the expression of cotton *PP2C* genes, thereby impairing ABA signal transduction (Figure 8C-b). In α-linolenic acid metabolism, differentially expressed genes involve intermediate product conversion-related enzymes such as lipoxygenase (*LOX2S*), hydroperoxide dehydratase (*AOS*), and allene oxide cyclase (*AOC*) (Figure 8D). Differentially expressed genes in the biosynthesis of flavonoids and phenylpropanolamine include chalcone synthase (*CHS*) and chalcone isomerase (*CHI*), which affect upstream metabolites in the flavonoid biosynthesis pathway, and cinnamonoyl-CoA reductase (*CCR*) and 4-coumarate-CoA ligase (*4CL*), which regulate the synthesis of phenylpropanolamine (Figure 8E). Four genes linked to cold stress were found. Through lipid metabolism, flavonoid biometabolism, and plant signal transduction, the *GhMDS11* gene may act as a regulatory factor in cold stress.

## 3. Discussion

Plant-specific transmembrane proteins are known as RLKs. They are known for having a wide range of types and locations. Their functions encompass the perception and transduction of external signals, which are integral to modulating plant development, hormonal responses, and defense mechanisms against environmental challenges. The *MDS* gene family represents one such group of RLKs. The regulatory functions of the *MDS* gene family in stress responses are not well characterized, despite a substantial body of literature on RLKs. The molecular mechanisms by which the *MDS* gene family regulates cold tolerance in cotton are still poorly understood. This study, based on the *MDS* gene family in *A. thaliana*, identified homologous genes of the *MDS* family in four cotton species. The combination of bioinformatic prediction, virus-induced gene silencing (VIGS), and transcriptome profiling enabled a deeper investigation into the spatiotemporal expression and stress-responsive regulation of *GhMDS* genes in cotton.

We identified 23–29 *MDS* members in diploid cotton, 40–42 *MDS* family members in tetraploid cotton, 27 members in closely related *T. cacao*, 4 members in *A. thaliana*, and 14 members in *O. sativa*. This indicates that the gene has undergone multiple rounds of duplication during evolution across species, including whole-genome duplication and tandem duplications (Figure 1). The evolutionary relationships of the cotton *MDS* gene family suggest that rapid expansion of *MDS* genes likely occurred after the divergence between monocots and dicots (Figure 2). The discovery of MDS1-CRCK3 binding and phosphorylation in planta points to MDS1 acting as a substrate for CRCK3 to amplify SUMM2 signaling cascades [19]. While retaining the core functions, some gene duplication forms functional redundancy, which can reduce the impact of a single gene mutation on the species and indirectly maintain the functional stability of gene families. In *A. thaliana*, the amino acid sequences of four *MDS* genes showed high similarity with their tissue-specific expression patterns [19]. Moreover, *mds1-−4* deletion mutants exhibited distinct traits under heavy metal ion stress only when all four *MDS* genes were deleted or mutated [24]. Therefore, in the subsequent experiment, several genes with significant gene expression under cold stress can be selected to knock out together in cotton plants, and the phenotypic conditions of cotton plants under stress can be compared and checked to speculate whether *GhMDS* gene also has redundancy phenomenon. The remarkable diversity exhibited by this protein family in physicochemical properties (Table S1) and genetic structure (Figure 3) likely results from long-term evolution to adapt to diverse functional requirements. We speculate that these gene members have undergone adaptive evolution under different environmental pressures, showing spatio-temporal or function-specific expression. Tissue-specific analysis reveals *GhMDS* is predominantly enriched in nutrient-rich tissues like roots, consistent with *MDS* gene expression patterns in *A. thaliana* [24]. The distribution and functions of different *MDS* genes are different in *A. thaliana* [19]. *AtMDS1* and *AtMDS3* show high expression in stomata and mid-vasicle sheaths. However, *mds1* mutant seedlings exhibit shortened main roots with reduced length and cell density in root meristems, while *mds3* mutants maintain wild-type root morphology but demonstrate reduced sensitivity to ABA-regulated stomatal movement [25]. Comprehensive analysis demonstrates that *GhMDS* gene members exhibit distinct expression patterns under stress conditions including cold, heat, salinity, drought, and pathogen infection (Figure 5), suggesting their crucial regulatory role in environmental adaptation. At present, no clear studies have reported the direct binding of transcription factors related to cold stress to the promoter region of CrRLKs. The promoter region of the *MDS* gene contains multiple *cis*-regulatory modules, including the jasmonic acid-responsive CGTCA motif, the abscisic acid-responsive ABRE, the antioxidant defense-related ARE, the low-temperature-responsive LTR, the drought-inducible MBS, and the light-responsive G-box. These modules collaboratively regulate cotton growth, its response to stress, and its reaction to light (Figure 4). This approach provides insights for elucidating the molecular processes that regulate stress responses. The expression levels of *GhMDS11* exhibited significant changes when induced by drought, salinity, cold, heat, and *V. dahliae* stress (Figure 5). Cotton plants with *GhMDS11* knockout induced by VIGS technology showed significantly reduced cold stress resistance compared to control groups: mutant plants exhibited more severe leaf wilting (Figure 7). The knockout plants displayed more severe leaf wilting, reduced antioxidant enzyme activities (superoxide dismutase SOD, catalase CAT, and peroxidase POD), and increased malondialdehyde (MDA) levels. Analysis of gene co-expression networks and transcriptomic data revealed that reduced *GhMDS11* expression disrupts multiple hormone signaling pathways. Specifically, suppressed expression of *PP2C* impairs ABA signaling transduction, while decreased levels of *LOX2S*, *AOS*, and *COI1* disrupt JA biosynthesis and signaling. The mechanism for making flavins is obstructed, and the pathways for making α-linolenic acid and linoleic acid are messed up. This hurts the cell membrane’s ability to eliminate reactive oxygen species and its structure (Figure 8). The α-linolenic acid metabolism serves as the precursor for jasmonic acid (JA) synthesis, while tryptophan metabolism forms the precursor for indole-3-acetic acid (IAA) production. Both pathways constitute core mechanisms in plant hormone synthesis. The key research focus lies in three critical pathways: Plant hormone signal transduction, MAPK signaling pathway in plants, and phenylpropanoid biosynthesis. These interconnected processes form a complete regulatory chain from “hormone synthesis → signal transduction → secondary metabolism.” These multi-level effects demonstrate that *GhMDS11* functions as a precision regulatory factor capable of precisely orchestrating transcriptome reprogramming during cold stress responses.

This study employed bioinformatics methods to analyze the phylogenetic relationships and structural characteristics of the GhMDS protein family, revealing its potential functions in cotton’s response to various stress types. VIGS experiments and transcriptome sequencing demonstrated that *GhMDS11* may coordinate physiological adaptations through interactions with hormonal and secondary metabolite pathways, playing a crucial role in cold resistance. Further exploration is needed to determine the specific regulatory targets of *GhMDS11* under low-temperature stress in cotton, its pathway interaction network, and whether this protein collaborates with other transcription factors or epigenetic modifiers to regulate downstream genes. Future research could utilize transgenic technology to develop genetically optimized lines and investigate functional redundancy and synergistic mechanisms among subfamily members, thereby providing theoretical foundations for developing crop stress-resistant breeding programs.

## 4. Materials and Methods

### 4.1. Plant Materials and Growth Conditions

The *G. hirsutum* cultivar ‘TM-1’ served as the main source of plant material for our investigation, and the seeds were kept in our laboratory. After being delinted with sulphuric acid, cotton seeds were steeped in distilled water for five hours. Following treatment, the seedlings were moved to seedling trays filled with a 3:1:1 mixture of nutrient-dense soil, perlite, and vermiculite. Under carefully monitored conditions, the plants were grown in nutrient pots with a photoperiod of 16 h of light and 8 h of darkness, and a constant temperature of 25 °C. Cotton seedlings were split into two groups at 15 days after germination. One group was kept at 25 °C (control), while the other group was moved to a growth chamber at 4 °C and exposed to light for 16 h [26]. For the time-course study, leaf tissues from the third and fourth true leaves were taken at 0, 1, 3, 6, 12, and 24 h after treatment [27,28]. For a subsequent total RNA extraction, the obtained samples were promptly flash-frozen in liquid nitrogen and kept at −80 °C.

### 4.2. Identification of MDS Gene Family Members

To create a local BLAST database, genomic information about *Gossypium* species was taken from the CottonMD database (https://yanglab.hzau.edu.cn/CottonMD) (accessed on 18 May 2025) [29]. It contained the *Gossypium* species listed below: *G. herbaceum* (WHU, A1), *G. hirsutum* (WHU, AD1), *G. raimondii* (HAU, D5), and *G. barbadense* (H7124, AD2). *A. thaliana* contains four *MDS* genes: *AtMDS1*, *AtMDS2*, *AtMDS3*, and *AtMDS4*. The reference sequence for the non-model species *T. cacao* was obtained from NCBI under the accession number GCA_000403535.1. The TAIR database (https://www.arabidopsis.org/) (accessed on 18 May 2025) provided the protein sequences of the four *AtMDS* genes, which were then used as query sequences to find homologous sequences in the genomic data of the four *Gossypium* species using BLASTP. To reduce false positives, an E-value threshold of 1.0 × 10^−20^ was applied, while other parameters were left at their default settings. The candidate sequences obtained from the search were submitted to the CDD database (http://www.ncbi.nlm.nih.gov/cdd/) (accessed on 21 May 2025) and MEME database (https://meme-suite.org/) (accessed on 21 May 2025) for identification of conserved domains in MDS proteins. Only sequences containing complete conserved domains were retained as members of the cotton *MDS* gene family, while redundant and fragmented sequences were excluded based on redundancy and sequence integrity. These *MDS* family genes were named according to their chromosomal locations. To assess basic physicochemical properties, the molecular weight and isoelectric point (pI) of each protein sequence were predicted with the ExPasy ProtParam online tool [30].

### 4.3. Phylogenetic Analysis of MDS Gene Family

Using the ClustalW algorithm in MEGA 11 software, all discovered *MDS* family member protein sequences from *Gossypium* spp., *O. sativa*, *A. thaliana*, and *T. cacao* were chosen for multiple sequence alignment. Using Maximum Likelihood (ML) approach, the phylogenetic tree was built while keeping all parameters set to their default settings. The itol web tool (https://itol.embl.de/) (accessed on 9 August 2025) was used to depict the evolutionary tree.

### 4.4. Chromosomal Localization and Synteny Analysis of MDS Gene Family

TBtools (v2.210) was used to extract the chromosomal location information of *MDS* family members from genomic annotation files, whilst genomic chromosome length information was acquired via the Fasta Stats module in TBtools. To evaluate gene duplication events and evolutionary relationships, the intra-genomic syntenic characteristics among *MDS* family members were analyzed using TBtools, with the results of gene alignment and chromosome localization graphically displayed through Circos (v1.9.x) software [31,32]. The analysis maintained all original parameters and configurations as specified in the methodology, ensuring comprehensive coverage of syntenic relationships within the cotton genome [33]. The visualization process employed standard color-coding schemes to differentiate between various chromosomal segments and their corresponding syntenic blocks, facilitating clear interpretation of the evolutionary patterns observed in the *MDS* family gene.

### 4.5. Analysis of Cis-Acting Elements in MDS Gene Family Promoters

We carried out a thorough examination of the cotton *MDS* gene family’s promoter regions. Using the PlantCARE database (http://bioinformatics.psb.ugent.be/webtools/plantcare/html/) (accessed on 23 May 2025), we extracted 2000 bp sequences upstream of the translation start sites from every identified *MDS* gene in order to predict *cis*-regulatory elements. TBtools was used to further classify and display the detected elements. In order to provide thorough coverage of potential regulatory motifs that might contribute to the transcriptional regulation of *GhMDS* genes under various circumstances, the analysis included all expected *cis*-acting regions without filtering. The visualization process employed standard color-coding schemes to differentiate between different categories of cis-regulatory elements, facilitating clear interpretation of their distribution patterns across the promoter regions of *GhMDS* family members.

### 4.6. Expression Analysis of GhMDS Genes

Transcriptome data of *GhMDS* across several tissues and under diverse abiotic stresses were acquired from the CottonMD website, whereas data pertaining to *GhMDS* under *V. dahliae* infection were sourced from the laboratory. This work investigated the expression patterns of *GhMDS* genes utilizing transcriptome data from various tissues and abiotic stress situations in *G. hirsutum*. Heatmaps were produced utilizing the Heatmap module within TBtools program [34].

Cotton plants with two leaves and one heart were put into a low-temperature incubator for 4 °C low-temperature growth. Three leaves from different plants were picked at 0, 1, 3, 6, 12, and 24 h and mixed. The Box RC401 RNA extraction kit was used to extract total RNA, and then cDNA synthesis was performed. Three biological replicates were used for the qRT-PCR tests, which were run with the following cycling parameters: 30 s of initial denaturation at 94 °C, 40 cycles of 94 °C for 5 s and 60 °C for 34 s, and a final extension at 72 °C for 34 s. *GhUBQ7* served as the internal reference for normalizing gene expression levels, which were then computed using the 2^−ΔΔCt^ technique [35,36]. All primers were designed using NCBI Primer-BLAST (Table S3). The expression analysis included all detected transcripts without any filtering to ensure comprehensive coverage of *GhMDS* gene expression patterns across different tissues and stress conditions. The heatmap visualization employed a standardized color gradient to represent relative expression levels, facilitating clear interpretation of expression variations among *GhMDS* family members.

### 4.7. Virus-Induced Gene Silencing (VIGS) Assay

VIGS is a reverse genetics method that uses the antiviral defense mechanisms of plants to quickly ascertain gene expression by using viral vectors encoding target gene segments to cause homologous mRNA to degrade or become methylated [37]. To validate the function of the *GhMDS11* gene, this study employed the tobacco rattle virus (TRV) system for VIGS experiments. A distinct leaf bleaching phenotype, indicative of successful gene silencing, was achieved by targeting the phytoene desaturase (*PDS*) gene, which served as a positive control for VIGS efficiency. The experimental procedure followed standard VIGS protocols with appropriate negative controls to ensure specificity of the observed phenotypes [38,39,40]. The TRV vectors containing target gene fragments were carefully designed to avoid off-target effects while maintaining optimal silencing efficiency. Primer 5 software was used to create primers with *Kpn* I and *Xba* I restriction sites for *GhMDS11* (Table S3). The Novozymes homologous recombination kit c112 was used to ligate the target gene fragment into the pTRV2 vector. *Agrobacterium tumefaciens* GV3101 was created by co-transforming the empty TRV2 vector, the recombinant TRV2:GhMDS11 vector, and the TRV1 vector. Five milliliters of LB liquid medium supplemented with rifampicin and kanamycin were used to inoculate individual colonies of successfully transformed *Agrobacterium*. We incubated these cultures overnight at 28 °C with shaking. For an overnight incubation period, 3 mL of the bacterial culture was then added to 50 mL of LB liquid medium that had been supplemented with rifampicin and kanamycin. After centrifuging the *Agrobacterium* cultures with various vectors for 10 min at 5000× *g* rpm, the bacterial pellets were resuspended in infiltration buffer (10 mM MgCl_2_, 200 μM acetosyringone, 10 mM MES) in order to achieve an OD_600_ of 1.0. To aid in the induction of acetosyringone, equal amounts of TRV1 and a number of TRV2 constructs (*TRV2:00*, *TRV2:GhMDS11*, or *TRV2:GhPDS*) were combined and incubated for three hours without light.

The *Agrobacterium* solution was administered to 8-day-old cotton seedlings possessing fully formed cotyledons using a 1 mL needleless syringe [41]. The infiltrated plants were moved to conventional growth chambers (16 h light, 8 h dark, 25 °C) after being incubated in darkness for 24 h at 22–25 °C [42]. Successful initiation of the VIGS system was confirmed by observing the characteristic bleaching phenotype in *TRV2:GhPDS*-infiltrated plants [43]. Leaves from both *TRV2:00* (negative control) and *TRV2:GhMDS11*-infiltrated plants were collected 20 days post-infiltration for RNA extraction and RT-qPCR analysis to evaluate silencing efficiency. Plants showing significantly reduced *GhMDS11* transcript levels were selected for subsequent experiments. The entire procedure maintained sterile conditions throughout the inoculation and cultivation processes to prevent microbial contamination, while all growth parameters were strictly controlled to ensure experimental consistency [44]. Phenotypic observations were conducted daily to monitor the progression of silencing effects, with particular attention paid to developmental changes in both control and silenced plants [45]. The selected plants with confirmed gene silencing were then subjected to detailed physiological and molecular analyses to investigate the functional consequences of *GhMDS11* suppression.

### 4.8. Data Statistical Analysis

All data were analyzed and visualized using SPSS software (version 25.0). Statistical significance, assessed by one- or two-way ANOVA, was defined as *p* < 0.05 (*) and *p* < 0.01 (**) [46]. Statistical analyses were conducted with appropriate Tukey’s post hoc test and independent-Sample *t*-test to verify the significance of observed differences between experimental groups. The variance homogeneity and normality assumptions were rigorously checked prior to performing ANOVA tests to ensure the validity of statistical conclusions [47]. The results were presented with exact *p*-values along with the corresponding significance markers to provide comprehensive statistical information. Error bars on graphical data denote the standard deviation (SD) or standard error of the mean (SEM), with the specific measure used corresponding to the information in the figure legends [48].

## 5. Conclusions

We performed a comprehensive analysis of the *MDS* gene family across four *Gossypium* species, which implicates these genes in the adaptation to cold stress. Gene expression profiling under biotic and abiotic stress conditions revealed significant differential expression of *GhMDS11*. VIGS experiments confirmed that *GhMDS11* enhances cold resistance in cotton. Transcriptome data indicated that *GhMDS11* likely improves cold tolerance by regulating cutin and wax biosynthesis, JA synthesis and signaling, ABA signal transduction, as well as zeatin and brassinosteroid biosynthesis pathways. This study deepens the molecular understanding of cotton’s stress response mechanisms and provides practical strategies for genetically improving cold tolerance in cotton through bioengineering approaches.

## Data Availability

The original contributions presented in this study are included in the article/Supplementary Material. Further inquiries can be directed to the corresponding author(s).

## Supplementary Materials

The following supporting information can be downloaded at: https://www.mdpi.com/article/10.3390/ijms262010144/s1.
