# Supplementary material for: Genome-Wide Characterization of the MDS Gene Family in Gossypium Reveals GhMDS11 as a Key Mediator of Cold Stress Response
Source: Int J Mol Sci. 2025 Oct 18;26(20):10144. doi: 10.3390/ijms262010144 (PMC12564768; doi:10.3390/ijms262010144)
Supplement: Supplementary file 1 [file ijms-26-10144-s001.zip › ijms-3897535-supplementary.pdf]

Supplementary Table S1: Physicochemical Property Analysis of the MDS Protein

Sequence ID	Number of Amino Acid	Molecular Weight	Theoretical pI	Instability Index	Aliphatic Index	Grand Average of Hydropathicity	Sub cellular Localization
Ghe07G23250	422	47546.29	5.72	43.58	91.23	-0.16	Plasma membrane
Ghe07G23270	414	47012.97	6.25	45.68	94.15	-0.185	Plasma membrane
Ghe07G23280	417	46970.84	6.02	47.59	91.15	-0.176	Plasma membrane
Ghe07G23490	869	97256.73	5.58	41.21	82.44	-0.205	Plasma membrane
Ghe07G23500	869	97134.34	5.51	40.27	81.9	-0.224	Plasma membrane
Ghe07G23520	548	61883.5	6.61	44.67	81.84	-0.179	Plasma membrane
Ghe07G23530	745	83587.45	6.14	36.21	85.41	-0.192	Plasma membrane
Ghe11G17800	349	38912.71	6.22	40.07	90.26	-0.001	Plasma membrane
Ghe11G17900	349	38926.62	6.13	37.79	92.75	0.021	Plasma membrane
Ghe11G18050	408	46501.41	6.19	45.01	90.15	-0.149	Plasma membrane
Ghe11G18070	381	42724.74	6.13	46.5	88.58	-0.159	Plasma membrane
Ghe11G18080	662	73841.79	6.63	36.34	89.85	-0.216	Plasma membrane
Ghe11G18090	719	80667.48	5.86	41.45	91.4	-0.108	Plasma membrane
Ghe11G18100	365	41153.11	7.57	52.4	87.1	-0.225	Plasma membrane
Ghe13G10560	813	91417.76	6.07	37.95	84.42	-0.212	Plasma membrane
Ghe11G18040	888	98335.03	6.75	41.46	82.41	-0.153	Plasma membrane
Ghe07G23190	794	89627.17	5.47	32.64	90.96	-0.083	Plasma membrane
Ghe07G09430	766	86829.31	8.82	38.19	83.25	-0.227	Plasma membrane
Ghe07G09450	741	83197.74	7.79	42.44	84.18	-0.206	Plasma membrane

Ghe03G16700	889	96969.55	5.55	37.17	83.46	-0.174	Plasma membrane
Ghe04G04590	854	95898.22	6.32	32.83	86.07	-0.205	Plasma membrane
Ghe10G15150	893	97913.45	5.87	37.87	81	-0.237	Plasma membrane
Ghe11G18020	915	101424.68	5.9	42.31	83.63	-0.201	Plasma membrane
Ghe09G27190	895	97793.38	5.67	38.64	82.58	-0.188	Plasma membrane
Ghe13G27170	921	102714.99	5.71	40.94	78.33	-0.264	Plasma membrane
Ghe03G24170	847	93517.2	6.94	33.81	82.18	-0.208	Plasma membrane
Ghe07G29810	890	98138	5.49	40.04	82.6	-0.216	Plasma membrane
Ghe07G23430	923	102871.42	6.04	43.76	78.86	-0.259	Plasma membrane
Ghe11G31680	841	94256.07	8.48	34.43	88.09	-0.101	Plasma membrane
Grai_01G005170	607	67345.4	7.3	42.13	89.59	-0.05	Plasma membrane
Grai_03G006780	788	87116.94	6.89	36.87	83.12	-0.224	Plasma membrane
Grai_03G012860	829	90372.92	5.55	38.86	82.69	-0.19	Plasma membrane
Grai_07G000810	889	98301.57	5.77	39.46	85.21	-0.208	Plasma membrane
Grai_07G006390	839	93817.7	6.75	39.68	84.8	-0.204	Plasma membrane
Grai_07G006400	809	90829.25	6.06	34.58	88.07	-0.155	Plasma membrane
Grai_07G006530	849	94993.63	5.29	39.35	78.41	-0.248	Plasma membrane
Grai_07G006570	868	96921.91	5.63	38.73	83.09	-0.235	Plasma membrane
Grai_07G006680	909	101597.91	5.79	43.14	78.36	-0.275	Plasma membrane
Grai_07G006830	794	89558.1	5.31	32.28	91.83	-0.061	Plasma membrane
Grai_07G021070	651	72153.04	8.19	41.33	89.08	-0.041	Plasma membrane
Grai_09G006930	631	71444.07	8.77	46.53	83.09	-0.247	Plasma membrane

Grai_09G021310	606	68376.41	7.2	41.26	85.07	-0.216	Plasma membrane
Grai_09G025520	895	97793.42	5.72	40.05	83.34	-0.187	Plasma membrane
Grai_10G013010	742	81242.64	5.82	37.8	80.8	-0.285	Plasma membrane
Grai_11G021700	657	73184.46	7.94	47.14	80.97	-0.23	Plasma membrane
Grai_11G021730	635	71531.74	7.7	50.4	84.85	-0.223	Plasma membrane
Grai_11G021750	627	70770.45	8.49	39.82	86.54	-0.115	Plasma membrane
Grai_11G022490	891	98729.58	6.42	41.09	87.17	-0.119	Plasma membrane
Grai_11G022500	908	100638.76	5.93	43.15	81.5	-0.207	Plasma membrane
Grai_13G001310	601	68707.51	7.75	41.55	80.62	-0.219	Plasma membrane
Grai_13G014170	851	94297.63	7.28	37.78	80.43	-0.282	Plasma membrane
Grai_13G027030	924	103477.51	6.6	38.87	80.82	-0.237	Plasma membrane
GB_A07G2072	493	55477.62	6.39	42.68	93.51	-0.2	Plasma membrane
GB_A07G2075	294	33206.09	5.64	45.63	95.14	-0.137	Plasma membrane
GB_A07G2089	677	75890.83	5.74	37.87	84.34	-0.198	Plasma membrane
GB_A07G2090	2144	240411.46	5.85	37.49	84.92	-0.198	Plasma membrane
GB_A11G1636	349	38870.67	6.37	40.07	89.97	0.008	Plasma membrane
GB_A11G1656	408	46515.4	6.07	45.64	89.19	-0.154	Plasma membrane
GB_A11G1658	381	42749.83	6.15	44.51	89.61	-0.155	Plasma membrane
GB_A11G1661	349	39277.19	6.28	44.5	95.82	-0.112	Plasma membrane
GB_A11G1662	376	42565.62	7.17	52.93	83.27	-0.267	Plasma membrane
GB_A13G0897	720	80949.85	5.75	36.6	84.74	-0.243	Plasma membrane
GB_D07G2078	416	47003.97	6.23	42.63	92.31	-0.169	Plasma membrane

GB_D07G2079	408	46041.81	5.99	43.02	94.63	-0.186	Plasma membrane
GB_D07G2132	868	97053.15	5.53	40.15	84.44	-0.215	Plasma membrane
GB_D07G2133	804	90181.8	6.09	36.54	81.73	-0.216	Plasma membrane
GB_D07G2721	640	72331.07	6.11	40.82	78.27	-0.274	Plasma membrane
GB_D11G1687	349	39016.67	5.95	41.2	89.68	-0.026	Plasma membrane
GB_D11G1695	339	37554.17	6.08	40.15	93.48	0.042	Plasma membrane
GB_D11G1707	194	21615.51	4.86	53.38	73.97	-0.259	Plasma membrane
GB_D11G1710	382	42972.01	6.13	44.89	89.35	-0.185	Plasma membrane
GB_D11G1711	1538	172747.06	6.37	40.96	89.91	-0.184	Plasma membrane
GB_D11G1712	368	41842.89	7.18	54.96	85.87	-0.245	Plasma membrane
GB_D13G0919	813	91840.27	6.1	38.85	85.38	-0.226	Plasma membrane
GB_D11G1706	887	98140.51	6.07	37.99	84.06	-0.146	Plasma membrane
GB_D07G2088	794	89497.02	5.35	32.73	91.34	-0.07	Plasma membrane
GB_A07G2066	794	89690.23	5.42	32.25	90.96	-0.077	Plasma membrane
GB_A11G1654	888	98339.1	6.75	42.49	83.5	-0.148	Plasma membrane
GB_A03G1035	889	97003.57	5.55	37.27	83.03	-0.176	Plasma membrane
GB_D02G1159	889	97013.56	5.42	37.17	83.58	-0.165	Plasma membrane
GB_A09G2437	895	97705.22	5.61	37.41	81.81	-0.181	Plasma membrane
GB_A10G1772	893	97850.43	6.06	37.57	81	-0.241	Plasma membrane
GB_A11G1653	915	101423.74	6.04	41.89	83.63	-0.202	Plasma membrane
GB_D11G1705	910	100858.99	6.26	41.39	81.43	-0.215	Plasma membrane
GB_D09G2283	895	97860.51	5.72	39.77	83.12	-0.181	Plasma membrane

GB_D07G0795	651	72173.02	8.02	41.62	88.63	-0.042	Plasma membrane
GB_A07G0788	652	72423.32	8.02	39.64	88.79	-0.037	Plasma membrane
GB_D07G2129	920	102693.17	5.56	40.31	78.58	-0.252	Plasma membrane
GB_D11G1767	562	63592.02	8.46	36.95	83.88	-0.216	Plasma membrane
GB_D07G2623	890	98282.6	5.83	39.81	85.76	-0.192	Plasma membrane
GB_D02G0606	848	93680.5	6.93	36.38	82.09	-0.207	Plasma membrane
GB_A02G0569	847	93616.34	7.11	34.97	82.18	-0.212	Plasma membrane
Ghi_A02G02081	847	93517.2	6.94	33.81	82.18	-0.208	Plasma membrane
Ghi_A03G05376	889	96969.55	5.55	37.13	83.46	-0.175	Plasma membrane
Ghi_A05G22511	854	95884.19	6.32	32.42	85.95	-0.208	Plasma membrane
Ghi_A07G04566	765	86438.07	7.12	38.14	80.43	-0.27	Plasma membrane
Ghi_A07G10906	794	89582.13	5.42	32.53	91.08	-0.079	Plasma membrane
Ghi_A07G10941	421	47465.3	5.75	41.02	92.38	-0.152	Plasma membrane
Ghi_A07G11001	919	102452.98	5.66	44.62	80.16	-0.219	Plasma membrane
Ghi_A07G11021	466	51676.19	5.78	38.15	86.39	-0.18	Plasma membrane
Ghi_A07G11026	345	38622.9	4.89	38.18	83.36	-0.249	Plasma membrane
Ghi_A09G13591	895	97676.21	5.66	36.71	82.47	-0.174	Plasma membrane
Ghi_A10G09396	883	96831.33	5.99	37.14	82.24	-0.231	Plasma membrane
Ghi_A11G08756	349	38884.7	6.37	40.07	89.97	0.008	Plasma membrane
Ghi_A11G08841	915	101324.64	6.04	42.02	83.63	-0.197	Plasma membrane
Ghi_A11G08846	888	98337.13	6.75	41.86	83.61	-0.148	Plasma membrane
Ghi_A11G08851	408	46537.45	6.19	45.64	89.19	-0.153	Plasma membrane

Ghi_A11G08861	381	42704.75	6.13	45.16	89.61	-0.156	Plasma membrane
Ghi_A11G08871	431	48291.81	5.35	44.28	90.44	-0.158	Plasma membrane
Ghi_A11G08876	376	42579.64	7.17	53.96	83.27	-0.267	Plasma membrane
Ghi_A13G04361	638	72527.68	6.68	40.02	84.06	-0.149	Plasma membrane
Ghi_A13G13111	909	101434.37	5.71	41.1	77.65	-0.292	Plasma membrane
Ghi_D02G02661	849	93730.56	6.93	36.1	82.33	-0.204	Plasma membrane
Ghi_D02G05531	889	96995.63	5.47	36.68	83.58	-0.166	Plasma membrane
Ghi_D04G01676	853	95565.68	5.97	33.42	86.74	-0.203	Plasma membrane
Ghi_D07G10276	794	89526.15	5.45	32.11	91.83	-0.063	Plasma membrane
Ghi_D07G10316	416	47003.97	6.23	42.63	92.31	-0.169	Plasma membrane
Ghi_D07G10356	907	101437.67	5.63	41.7	78.73	-0.275	Plasma membrane
Ghi_D07G10376	868	97044.14	5.5	40.46	84.44	-0.216	Plasma membrane
Ghi_D07G10381	869	97100.09	5.37	36.55	79.98	-0.235	Plasma membrane
Ghi_D07G10401	847	95011.7	5.35	37.95	80.2	-0.273	Plasma membrane
Ghi_D07G12881	890	98298.61	5.77	40.56	85.55	-0.195	Plasma membrane
Ghi_D09G10016	895	97818.43	5.72	39.56	82.79	-0.184	Plasma membrane
Ghi_D10G05856	883	96806.35	5.87	36.41	82.36	-0.215	Plasma membrane
Ghi_D11G08531	339	37554.17	6.08	40.15	93.48	0.042	Plasma membrane
Ghi_D11G08591	910	100695.73	6.08	41.59	81.43	-0.214	Plasma membrane
Ghi_D11G08596	887	98168.56	6.07	37.99	84.27	-0.143	Plasma membrane
Ghi_D11G08606	382	43264.11	6.33	45.09	93.46	-0.043	Plasma membrane
Ghi_D11G08616	382	43046.03	6.13	45.4	88.06	-0.215	Plasma membrane

Ghi_D11G08621	612	68368.3	5.15	37.06	90.67	-0.116	Plasma membrane
Ghi_D11G08626	379	42444.71	6.11	42.33	89.79	-0.15	Plasma membrane
Ghi_D11G08631	386	43896.24	6.32	41.74	90.67	-0.183	Plasma membrane
Ghi_D11G08636	369	41895.94	7.19	53.02	86.42	-0.24	Plasma membrane
Ghi_D13G04651	832	93929.67	6.07	39.73	85.07	-0.206	Plasma membrane

Supplementary Table S2: Renaming of the *MDS* Gene Family

**Sequence ID**	**Gene ID**	**Sequence ID**	**Gene ID**	**Sequence ID**	**Gene ID**
Ghe03G16700	GheMDS1	Grai_11G021730	GrMDS17	GB_D11G1767	GbMDS39
Ghe03G24170	GheMDS2	Grai_11G021750	GrMDS18	GB_D13G0919	GbMDS40
Ghe04G04590	GheMDS3	Grai_11G022490	GrMDS19	Ghi_A02G02081	GhMDS1
Ghe07G09430	GheMDS4	Grai_11G022500	GrMDS20	Ghi_A03G05376	GhMDS2
Ghe07G09450	GheMDS5	Grai_13G001310	GrMDS21	Ghi_A05G22511	GhMDS3
Ghe07G23190	GheMDS6	Grai_13G014170	GrMDS22	Ghi_A07G04566	GhMDS4
Ghe07G23250	GheMDS7	Grai_13G027030	GrMDS23	Ghi_A07G10906	GhMDS5
Ghe07G23270	GheMDS8	GB_A02G0569	GbMDS1	Ghi_A07G10941	GhMDS6
Ghe07G23280	GheMDS9	GB_A03G1035	GbMDS2	Ghi_A07G11001	GhMDS7
Ghe07G23430	GheMDS10	GB_A07G0788	GbMDS3	Ghi_A07G11021	GhMDS8
Ghe07G23490	GheMDS11	GB_A07G2066	GbMDS4	Ghi_A07G11026	GhMDS9
Ghe07G23500	GheMDS12	GB_A07G2072	GbMDS5	Ghi_A09G13591	GhMDS10
Ghe07G23520	GheMDS13	GB_A07G2075	GbMDS6	Ghi_A10G09396	GhMDS11
Ghe07G23530	GheMDS14	GB_A07G2089	GbMDS7	Ghi_A11G08756	GhMDS12
Ghe07G29810	GheMDS15	GB_A07G2090	GbMDS8	Ghi_A11G08841	GhMDS13
Ghe09G27190	GheMDS16	GB_A09G2437	GbMDS9	Ghi_A11G08846	GhMDS14
Ghe10G15150	GheMDS17	GB_A10G1772	GbMDS10	Ghi_A11G08851	GhMDS15
Ghe11G17800	GheMDS18	GB_A11G1636	GbMDS11	Ghi_A11G08861	GhMDS16
Ghe11G17900	GheMDS19	GB_A11G1653	GbMDS12	Ghi_A11G08871	GhMDS17
Ghe11G18020	GheMDS20	GB_A11G1654	GbMDS13	Ghi_A11G08876	GhMDS18
Ghe11G18040	GheMDS21	GB_A11G1656	GbMDS14	Ghi_A13G04361	GhMDS19
Ghe11G18050	GheMDS22	GB_A11G1658	GbMDS15	Ghi_A13G13111	GhMDS20
Ghe11G18070	GheMDS23	GB_A11G1661	GbMDS16	Ghi_D02G02661	GhMDS21
Ghe11G18080	GheMDS24	GB_A11G1662	GbMDS17	Ghi_D02G05531	GhMDS22
Ghe11G18090	GheMDS25	GB_A13G0897	GbMDS18	Ghi_D04G01676	GhMDS23
Ghe11G18100	GheMDS26	GB_D02G0606	GbMDS19	Ghi_D07G10276	GhMDS24
Ghe11G31680	GheMDS27	GB_D02G1159	GbMDS20	Ghi_D07G10316	GhMDS25
Ghe13G10560	GheMDS28	GB_D07G0795	GbMDS21	Ghi_D07G10356	GhMDS26
Ghe13G27170	GheMDS29	GB_D07G2078	GbMDS22	Ghi_D07G10376	GhMDS27
Grai_01G005170	GrMDS1	GB_D07G2079	GbMDS23	Ghi_D07G10381	GhMDS28
Grai_03G006780	GrMDS2	GB_D07G2088	GbMDS24	Ghi_D07G10401	GhMDS29
Grai_03G012860	GrMDS3	GB_D07G2129	GbMDS25	Ghi_D07G12881	GhMDS30
Grai_07G000810	GrMDS4	GB_D07G2132	GbMDS26	Ghi_D09G10016	GhMDS31
Grai_07G006390	GrMDS5	GB_D07G2133	GbMDS27	Ghi_D10G05856	GhMDS32
Grai_07G006400	GrMDS6	GB_D07G2623	GbMDS28	Ghi_D11G08531	GhMDS33
Grai_07G006530	GrMDS7	GB_D07G2721	GbMDS29	Ghi_D11G08591	GhMDS34
Grai_07G006570	GrMDS8	GB_D09G2283	GbMDS30	Ghi_D11G08596	GhMDS35
Grai_07G006680	GrMDS9	GB_D11G1687	GbMDS31	Ghi_D11G08606	GhMDS36
Grai_07G006830	GrMDS10	GB_D11G1695	GbMDS32	Ghi_D11G08616	GhMDS37
Grai_07G021070	GrMDS11	GB_D11G1705	GbMDS33	Ghi_D11G08621	GhMDS38
Grai_09G006930	GrMDS12	GB_D11G1706	GbMDS34	Ghi_D11G08626	GhMDS39

Grai_09G021310	GrMDS13	GB_D11G1707	GbMDS35	Ghi_D11G08631	GhMDS40
Grai_09G025520	GrMDS14	GB_D11G1710	GbMDS36	Ghi_D11G08636	GhMDS41
Grai_10G013010	GrMDS15	GB_D11G1711	GbMDS37	Ghi_D13G04651	GhMDS42
Grai_11G021700	GrMDS16	GB_D11G1712	GbMDS38		

Supplementary Table S3: *GhMDS* family fluorescence quantification and VIGS primer table

Gene ID	Forward Primer (5'-3')	Reverse Primer (5'-3')
GhMDS03	CCCACATCTCGTCTCCCTCATC	CGTGATCCTTGCCAATCCCAAAG
GhMDS04	CGCAGAAGAACGACCATCCATG	GACCCCGCTGTGAGGAATTATAC
GhMDS07	TCTTAGTGCGTTCCGAACTGAAG	GATTGTTTGCCCCTTCTCATTGC
GhMDS09	AAGGGCAGGATAGCAGCAGAG	TCTAGCATCACCTCCACTTCACC
GhMDS11	GCCGCCACCAACAACTTCC	CGCTTGACAACAACTATGGTTCC
GhMDS23	GCCTATTGTTGTGTTTGCCAGAC	CGCTCTCCTTCTCCGAATCAG
GhMDS24	GGCAGTTCAGGAGGAGGAGTAAG	AGGCAGAACCCGATAAGAGAGAC
GhMDS26	TGAGGTGGAGGTGACATTGGAG	GTGTTCTGGGAGCGACGAATG
GhMDS32	ATGGATCATGTCAAGACGCAGTG	CGCTCTTCGCAAATCCCAATAAG
VIGS-GhMDS11	aaggttaccgaattctctagaATGTCTGTTATAACTTTTGTGTCCTTGC	gagacgcgtgagctcggtaccTCAACGCCCTTTAGGGTTCA

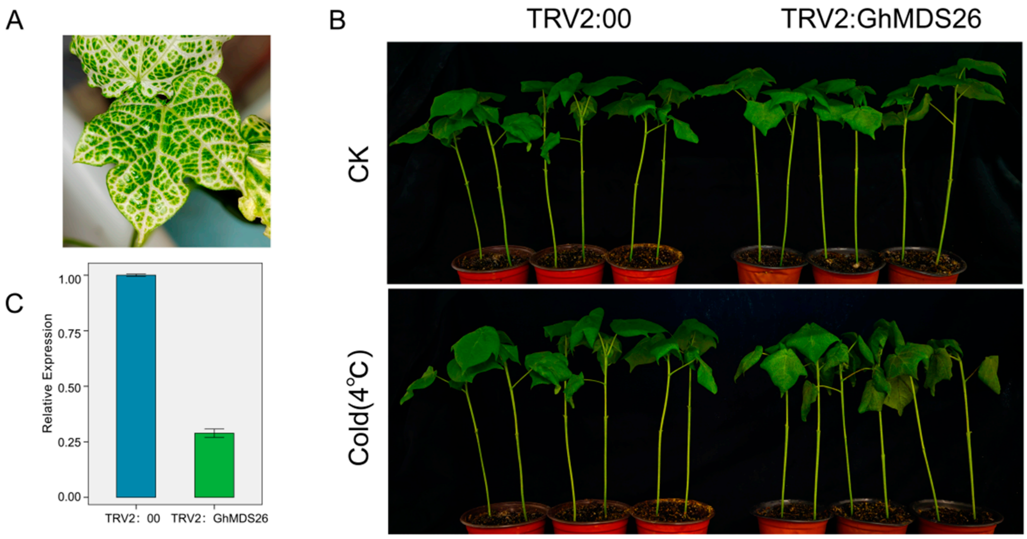

Supplementary Figure S1: Impact of VIGS-mediated *GhMS26* silencing on cotton's ability to withstand cold. (A) Phenotype of positive control *TRV2:PDS*; (B) Phenotype of *GhMDS26*-silenced *G. hirsutum* plants after cold stress treatment; (C) RT-qPCR analysis of *GhMDS26* gene expression level changes in cotton leaves.

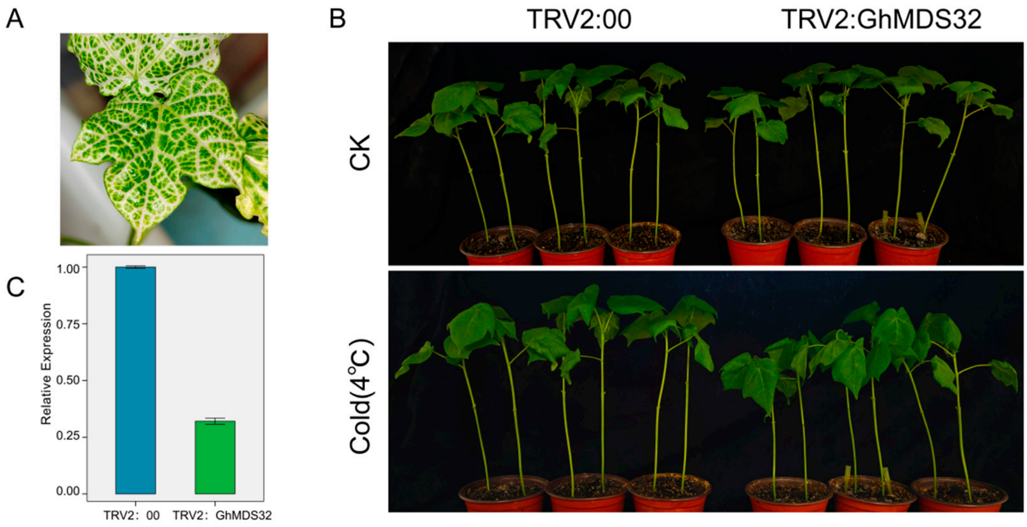

Supplementary Figure S2: Impact of VIGS-mediated *GhMS32* silencing on cotton's ability to withstand cold. (A) Phenotype of positive control *TRV2:PDS*; (B) Phenotype of *GhMDS11*-silenced *G. hirsutum* plants after cold stress treatment; (C) RT-qPCR analysis of *GhMDS32* gene expression level changes in cotton leaves.

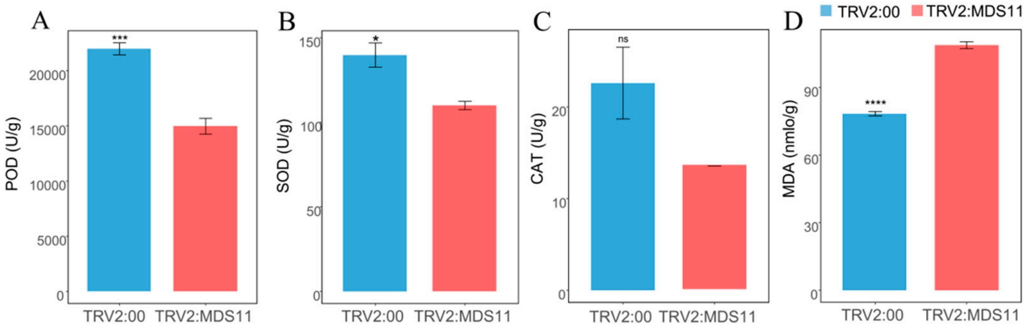

Supplementary Figure S3: Changes in physiological indices of gene-silenced plants after cold stress treatment. (A-D) The changes of POD, SOD, CAT and MDA were studied after cold treatment in genetic silent cotton. The data were analyzed by independent-Sample T-test of SPSS software. *** $p < 0.001$; ** $p < 0.01$; * $p < 0.05$.
